# Assessment of the eye surface and subjective symptoms after using 0.1% dexamethasone drops with and without preservatives in patients after cataract surgery

**DOI:** 10.1038/s41598-023-44939-1

**Published:** 2023-10-30

**Authors:** Maria Muzyka-Woźniak, Łukasz Stróżecki, Joanna Przeździecka-Dołyk

**Affiliations:** 1 Ophthalmology Clinical Centre SPEKTRUM, Research and Development Centre CREO, Zaolziańska 4 Str., 53-334 Wrocław, Poland; 2 Department of Anaesthesiology and Intensive Therapy, Wroclaw Medical University, Wybrzeże Ludwika Pasteura 1, 50-367 Wrocław, Poland; 3 Department of Optics and Photonics, Wroclaw University of Science and Technology, Wybrzeże Wyspiańskiego 27, 50-370 Wrocław, Poland

**Keywords:** Outcomes research, Rehabilitation

## Abstract

Cataract surgery can cause dry eye symptoms. One of the many factors compromising the ocular surface is the use of benzalkonium chloride (BAC)-preserved topical eye drops administered during the postoperative period. In this open-label, prospective, randomized, comparative clinical trial, 40 patients not previously affected by dry eye disease were assigned to receive either preservative-free (PFD) or preserved (PD) dexamethasone 0.1% eye drops for two weeks after a standard phacoemulsification procedure. Fluorescein break-up time, ocular surface staining score, Schirmer test, Ocular Surface Disease Index and anterior chamber (AC) cells were evaluated at baseline prior to the surgery and 2 weeks after surgery. No statistically significant differences in baseline assessments were observed between groups. At week 2, a significant increase in corneal staining scores (p = 0.003) and foreign body sensation (p = 0.04) was observed for the PD group only. The conjunctival staining score was significantly higher in both groups. The mean AC cell grading was higher in the PFD group than in the PD group (0.28 ± 0.30 and 0.07 ± 0.18, respectively; p = 0.013). Preservative-free dexamethasone eye drops after cataract surgery caused milder dry eye symptoms as compared with preserved dexamethasone. The AC inflammation control comparison may require a larger study group. Trial registration: ClinicalTrials.gov identifier NCT05753787, 03/03/2023.

## Introduction

Despite being a low-risk, high-volume and quick ambulatory procedure, modern cataract surgery can cause adverse dry eye symptoms, diminishing the positive effects of surgical treatment^1^. The incidence of postoperative dry eye signs and symptoms is high, ranging from 9 to 100% and lasting up to 6 months in some patients^2–6^. There are many possible factors involved in ocular surface disease after phacoemulsification, including transection of the corneal nerves, phototoxicity, repeated irrigation of the ocular surface during surgery, elevation of inflammatory cytokines, the use of iodine povidone, topical anaesthesia and antibiotics as well as preservatives containing topical eye drops administered after surgery^2–6^. Each of the possible causes should be dealt with care, and its effect should be properly addressed to achieve the best postsurgical outcomes in terms of surgical success as well as patient-oriented well-being and subjective comfort.

Benzalkonium chloride (BAC), used as a preservative in many eye drops, induces tear film instability and ocular surface damage in a dose-dependent manner^7^. Preserved prednisolone used for the treatment of acute anterior uveitis can cause higher toxicity and subjective discomfort than preservative-free methylprednisolone^8^. Surprisingly, there are no studies targeting the influence of preserved and preservative-free dexamethasone on the ocular surface after cataract surgery. There have been reports on other steroids, such as prednisolone, fluorometholone or dexamethasone/antibiotic fixed combination, with or without nonsteroidal anti-inflammatory drops (NSAID), none of them exclusively examining the effects of preservatives in dexamethasone formulation after cataract surgery on the ocular surface^8–11^.

The goal of this interventional, real-world data study is to evaluate the impact of preserved and preservative-free 0.1% dexamethasone eyedrops on subjective and objective dry eye disease (DED) signs and symptoms after cataract surgery.

## Materials and methods

### Study design

This open-label, prospective, randomized, investigator-masked, real-world data, comparative clinical study was conducted from November 2020 to December 2021 at the SPEKTRUM Ophthalmology Clinical Centre. The study was conducted in accordance with the Good Clinical Practices and the tenets of the Declaration of Helsinki and approved by the Bioethics Committee of Wroclaw Medical University (KB 272/2017). The study was registered in the clinical trials by the number NCT05753787 (03/03/2023). All patients gave written informed consent to the study and to the surgery.

The main outcome measures were fluorescein break-up time (FBUT), ocular surface staining, OSDI and subjective symptoms of DED evaluated before and 2 weeks after cataract surgery. The secondary outcome measures were anterior chamber (AC) cells grade and intraocular pressure assessed 2 weeks after surgery.

### Study population

The study included cataract patients who qualified for cataract surgery according to the Polish Standards of Care and Guidelines with absent or mild dry eye disease. The general exclusion criteria were diabetes, allergy, autoimmune diseases, including rheumatoid arthritis, use of systemic treatment that can cause DED, such as antidepressants, antihistamines and spasmolytics, use of systemic or local corticosteroids or nonsteroidal anti-inflammatory drugs. The ocular exclusion criteria at baseline were as follows: OSDI > 15, ocular staining score > 1.0, FBUT < 5 s, Schirmer test < 10 mm after 5 min, use of any other eyedrops other than those included in the study protocol, glaucoma, any previous ocular surgery, pseudoexfoliation syndrome, very advanced cataract (> 4 LOCS III), and endothelial count number below 2000 cells/mm^2^. Patients with any intraoperative or postoperative complications were withdrawn from the study.

Patients were randomly assigned to one of the treatment groups: the PFD group received preservative-free 0.1% dexamethasone disodium phosphate eyedrops (Dexaprotect, S-Lab, Mirków, Poland) 4 times daily for 2 weeks; the PD group received 0.1% dexamethasone disodium phosphate eyedrops preserved with BAC (Dexamethasone WZF 0,1%, Polfa, Warsaw, Poland) 4 times daily for 2 weeks after surgery. The parallel assignment to one of the study groups followed the random allocation rule, with block size two and four. The random allocation sequence was generated by LS. The investigator (MMW) was blinded to the assigned treatment. The composition of both dexamethasone formulations is presented in Table 1. Additionally, both groups received the same standard postoperative treatment: moxifloxacin 0.5% (Vigamox 5 mg/ml, Alcon Laboratories, Inc. Fort Worth, Texas, USA) 4 times daily for one week after surgery and tear film substitution with the same formulation containing dexpanthenol 2%, hydroxypropylcellulose 0.5% and polyhexanide 0.00001% (Dexoftyal, Verco S.A., Warsaw, Poland) 3 times daily. The medications were dispensed as an open-label treatment.

**Table 1:** The composition of the studied dexamethasone 0.1% formulations. *PFD* preservative-free dexamethasone, *PD* preserved dexamethasone.

	PFD (preservative-free dexamethasone) group	PD (preserved dexamethasone) group
Active compound	Dexamethasone sodium phosphate	Dexamethasone disodium phosphate
Concentration	1 mg/ml	1 mg/ml
Formulation	Water solution	Suspension
Additives	Disodium phosphate dodecahydrate Natrium chloride Edetate disodium Hydrochloric acid Sodium hydroxide Aqua pro iniectione	Disodium phosphate dodecahydrate Sodium dihydrogen phosphate monohydrate Natrium chloride Edetate disodium Benzalkonium chloride Polysorbate Ethyl alcohol 96% Sodium hydroxide Purified water

Only one eye per patient was analysed. If the other eye was to be operated on, the surgery was scheduled after completion of all study assessments.

### Assessments

All assessments were performed prior to the surgery (baseline) and 15 (± 2) days (week 2) after surgery in the same, standardized order according to the TFOS DEWS II recommendations^12^. The assessments included the Ocular Surface Disease Index, subjective symptoms recording, fluorescein tear break-up time (FBUT), ocular staining with fluorescein and lissamine green assessed with the Ocular Staining Score, Schirmer test 1 without anesthesia and intraocular pressure (I-Care tonometer)^13,14^. OSDI was completed by the subject without assistance. Subjective symptoms such as itching, discomfort, foreign body sensation, photophobia and tearing were asked and graded depending on severity on a four-step scale (0–3), provided in the Supplementary Materials (Table S1). Additionally, at the week 2 visit, the AC cells grade was recorded. The intensity of the AC cellular reaction was assessed based on the number of inflammatory cells seen in a 1 × 1-mm full intensity beam at 45 to 60 degrees of the biomicroscope and was graded according to the SUN Working Group Classification (Table S2)^15^. Other preoperative and postoperative clinical evaluations were performed as a standard cataract surgery procedure.

### Surgical procedure

For all patients, standard phacoemulsification under topical anaesthesia (lidocaine 4%) was performed via a temporal 2.2 mm posterior limbal incision. The following eyedrops were administered 20 min before surgery: 1% tropicamide, 10% neosynephrine, nepafenac and moxifloxacin. Standard antisepsis of the ocular surface with 5% iodine povidone for 3 min and periocular skin with 10% iodine povidone for 5 min was performed immediately before draping the eyelids. At the time of surgery, all patients received intracameral cefuroxime (Aprocam, Théa Pharmaceuticals Limited, Newcastle-under-Lyme, UK) injection.

### Sample size

Assuming a 1:1 ratio between the PFD group and PD group with the target difference for FBUT set at 1.8 ± 2.0 s, the power of the study was estimated to be 80% with a *p* value of 0.05 and 20 subjects in each group. To allow for 20% patient dropout, 48 eyes of 48 patients were included.

### Statistical analysis

Data were calculated as the mean ± standard deviation (SD). The confidence interval was determined at 95% confidence level. Between-group comparisons of age, Schirmer test, FBUT, ocular surface staining, OSDI scores, IOP and phacoemulsification time were performed using the Mann–Whitney *U* test with continuity correction. Within-group comparisons were made using the Wilcoxon matched-pairs test. For the categorical variables, the Chi-square (χ^2^) goodness of fit test was performed for between- and within-group comparisons. A p-value lower than 0.05 was considered statistically significant. Statistical analyses were performed using the software package Statistics 13 (StatSoft Poland).

## Results

### Patients

Forty-six patients to undergo planned cataract surgery with absent or mild dry eye disease were randomly assigned to receive either nonpreserved (PFD group) or preserved (PD group) dexamethasone eye drops in postoperative treatment. Six patients were lost to follow-up, leaving 20 patients in each group, with 9 left eyes and 11 right eyes in each group. Seventeen patients (80%) in the PFD group and 12 (60%) patients in the PD group were female. The mean ages in the PFD and PD groups were 68 ± 6.7 and 65 ± 9.1 years, respectively (p = 0.37). No statistically significant differences in baseline subjective and objective assessments were observed between the PFD and PD groups (Table 2). Specifically, there were no significant differences between the 2 examined groups for baseline subjective dry eye symptoms (itching, foreign body sensation, photophobia and tearing), with the majority of patients reporting 0 or 1st grade of symptoms (p > 0.18, Pearson’s Chi-squared test). No intra- or postoperative complications were observed. The mean effective phacoemulsification time was 5.07 ± 3.4 s in the PFD group and 3.89 ± 3.4 s in the PD group (p = 0.15, Mann–Whitney *U* test).

**Table 2:** Baseline clinical characteristics in the PFD group (preservative-free dexamethasone) and PD group (preserved dexamethasone) presented as the mean and standard deviation.

	PFD (n = 20)	PD (n = 20)	p*
OSDI (score)	5.9 ± 7.3	6.9 ± 7.9	1.0
FBUT (s)	10.2 ± 5.3	9.9 ± 3.6	0.86
Fluoresceine staining (score)	0.2 ± 0.4	0.2 ± 0.4	0.63
Lissamine Green (score)	0.05 ± 0.22	0.0 ± 0.2	0.79
Schirmer test (mm)	12.6 ± 7.3	14.2 ± 6.3	0.35
IOP (mmHg)	17.7 ± 2.7	17.7 ± 1.9	0.82

*Mann–Whitney *U* test with continuity correction.

### Postoperative dry eye assessments

There was a significant increase in fluorescein corneal staining in the PD group at the week 2 visit compared to baseline. No significant change in fluorescein staining in the PFD group was observed. Both groups showed a significant increase in lissamine green conjunctival staining (Table 3). FBUT at visit 2 was shorter for all patients compared to baseline; however, this change was not significant for PFD patients and was borderline statistically significant for PD patients (p = 0.05, Wilcoxon signed-rank test).

**Table 3:** The change between preoperative (baseline) and postoperative (week 2) mean measures and 95% confidence intervals in the PFD (preservative-free dexamethasone) and PD (preserved dexamethasone) groups (Wilcoxon signed-rank test). Significant differences with p < 0.05 are marked with *.

	PFD group			PD group		
	Baseline Mean (95% CI)	Week 2 Mean (95% CI)	p	Baseline Mean (95% CI)	Week 2 Mean (95% CI)	p
Fluorescein staining (score)	0.2 (0.02–0.38)	0.57 (0.27–0.87)	0.09	0.15 (0.0–0.36)	0.85 (0.50–1.20)	0.003*
OSDI (score)	5.9 (2.6–9.2)	5.19 (3.30–7.08)	0.9	6.90 (3.43–10.37)	5.22 (1.43–9.01)	0.42
FBUT (s)	10.18 (7.81–12.55)	8.35 (6.16–10.54)	0.21	9.93 (8.36–11.5)	7.51 (5.65–9.37)	0.05
Lissamine Green (score)	0.05 (0.0–0.15)	0.84 (0.22–1.46)	0.02*	0.05 (0.0–0.14)	1.0 (0.51–1.49)	0.005*
Schirmer test (mm)	12.6 (9.40–15.8)	10.52 (7.72–13.32)	0.07	14.25 (11.52–16.98)	10.85 (7.68–14.02)	0.09
IOP (mmHg)	17.75 (16.57–18.93)	14.68 (13.39–15.97)	< 0.001*	17.7 (16.85–18.55)	16.8 (15.47–18.33)	0.11

There was no significant change between pre- and postoperative subjective symptoms such as itching, photophobia and tearing in either group, except foreign body sensation, which was significantly greater at week 2 in the PD group (p = 0.04, Pearson’s Chi-square).

At week 2, a significant lowering of IOP in the PFD group was observed (p = 0.0005, Wilcoxon signed rank-test), while in the PD group, there was no change in IOP compared to baseline. Between-group comparisons showed a significantly lower IOP in the PFD group at week 2 (p = 0.04, Mann–Whitney *U* test). The change between preoperative and postoperative measures in the PFD and PD groups is summarized in Table 3.

The mean AC cells grading was higher in the PFD group (0.28 ± 0.30) than in the PD group (0.07 ± 0.18) at the week 2 visit (p = 0.013, Mann–Whitney *U* test). The grade of AC cells in the PFD group was never higher than + 0.5 (1–5 cells in field), except for one eye with grade 1 AC cells (6–15 cells in field).

## Discussion

Dexamethasone as topical treatment is routinely used after cataract surgery. BAC-containing eyedrops are one of the main factors contributing to iatrogenic postcataract dry eye disease^16,17^. In this prospective study, we included patients not previously affected by DED who underwent phacoemulsification for senile cataract and who were randomly assigned to dexamethasone eye drops with or without BAC. In particular, our purpose was to evaluate the effects of preserved and nonpreserved dexamethasone on ocular signs and symptoms of DED after cataract surgery.

We found that the group receiving preserved dexamethasone eye drops had more conjunctival and corneal staining as well as foreign body sensation at 2 weeks after cataract surgery in comparison to patients receiving nonpreserved formulation. Another study assessed netylmicin/dexamethasone preservative-free and tobramycin/dexamethasone-preserved fixed combinations after cataract surgery and found no significant differences in ocular surface status^9^. Jee et al. showed improvement of dry-eye symptoms and signs in patients with pre-existing dry-eye syndrome after cataract surgery in patients treated with preservative-free fluorometholone 0.1% and preservative-free sodium hyaluronate 0.1% compared to patients treated with preserved medications^11^. In our study, patients received the same postoperative treatment regimen with the only discriminant being the presence or absence of BAC in the dexamethasone formulation.

Benzalkonium chloride (BAC) induces tear film instability and ocular surface damage^7^. Specifically, it can destroy the tight junctions that are located in the superficial cells of the corneal epithelium and lead to apoptosis of the conjunctival epithelium^18,19^. Additionally, BAC can stimulate the overexpression of inflammatory mediators in the epithelium, such as ICAM-1 or interleukins IL-6, IL-8, and IL-10^20,21^. This can induce the loss of goblet cells and reduce mucin production, leading to a shorter tear film break-up time^22^. Our study shows that corneal fluorescein staining was significantly increased in the group receiving BAC-preserved dexamethasone, and the FBUT in this group was shorter, bordering statistical significance (p = 0.05). In contrast, these changes were not significant in the group receiving nonpreserved dexamethasone. In the presented context, we confirmed that preservatives are the cause of corneal epithelium damage as well as changes in the tear film. This is an important outcome that guides surgeons to care for the patient’s ocular surface after surgery, as it may be an important factor for patient well-being.

Another possible explanation for the more pronounced staining and ocular discomfort in the preserved dexamethasone group is the effect of the suspension formula on the ocular surface. Suspensions contain microparticles of the active substance, which may cause microabrasions of the corneal epithelium^23^. According to Schoenwald and Stewart, a smaller mean particle size in 0.1% dexamethasone suspensions resulted in greater drug levels in the cornea and aqueous humor^24^. However, particles that are too small may undergo faster elimination from the conjunctival sac; therefore, there is a lower limit for particle-size retention and an upper limit for ocular surface irritation^25^. Consequently, the active substance water solution may also be characterized by shorter retention in the conjunctival sac and fewer negative effects on the ocular surface. However, the shorter retention may be responsible for worse penetration.

Administration of dexamethasone sodium phosphate is subjected to a hydrolysis reaction catalysed by enzymes in the tear film and cornea, partly converting water-soluble phosphate into lipid-soluble dexamethasone alcohol, which theoretically penetrates the lipophilic corneal epithelium more easily^26^. Concerning AC cellular reaction, both groups showed very low cellular counts at the 2-week visit, with 1–5 cells in the field (grade + 0.5 according to SUN). However, eyes receiving nonpreserved dexamethasone had a significantly higher mean AC cells score within this low grade (0.28 versus 0.07, p = 0.013). Clinically, this difference may be regarded as irrelevant because zero-to-trace (+ 0.5) anterior chamber cells are used in other clinical trials as one category for inflammation control following cataract surgery^27^. A possible explanation for slightly better inflammation control in the PD group is that dexamethasone suspension penetrates the cornea better than water solution. Dexamethasone suspension gives concentrations of steroids in the aqueous humor approximately three times higher than with dexamethasone solution drops^28^. In our study, patients did not receive topical nonsteroidal anti-inflammatory drugs, which presumably would have obscured this difference, as in a study by Cagini, who did not observe any difference in aqueous humour flare values^29^. Nevertheless, in our study, the AC cellular reaction was very low in both examined groups. Interestingly, the PD group had a relatively higher IOP after surgery, which may be attributed to the stronger steroid effect of preserved dexamethasone on ocular tissues. A larger sample size is needed to confirm this observation.

We chose the 2-week time point for our assessments, presuming that at this time, all short-term ocular surface damage caused by the procedure itself (corneal cuts, iodine povidone irritation, speculum trauma) should be healed, leaving the postoperative eye drops as a main cause of any dry eye symptoms, with their peak intensity at that time^30^. Jee et al. reported that at one and two months after surgery, treatment with preservative-free sodium hyaluronate 0.1% and fluorometholone 0.1% eyedrops actually improved the OSDI score, TBUT, Schirmer I score, corneal staining, and impression cytological findings compared to treatment with preserved eyedrops in patients with pre-existing dry-eye syndrome^11^. Our study compared a different steroid, dexamethasone, and the assessments were performed much earlier, 2 weeks after surgery. This explains why in our study, slightly shorter FBUTs and lower Schirmer test results were recorded in both groups. This difference in assessment time-point is even more visible for corneal staining, which was significantly worse in our group treated with preserved dexamethasone, while in the cited study, the staining was significantly decreased one and two months postop in comparison to baseline preoperative status. The time-point for assessments is crucial, since most of the dry eye symptoms induced by cataract surgery return to almost the preoperative level 1 month postoperatively^31^.

In the paper by Kohli, an OSDI score > 33 was reported in 32% of patients 2 weeks after cataract surgery^32^. Other studies also show a transient increase in the OSDI score, with a different time lapse to return to the baseline level, ranging from 1 to 3 months^33–35^. In our cohort of non-dry eye patients, OSDI evaluated 2 weeks postop remained at the preoperative low level. This may be explained by the routine use of artificial tear formulations, which are standard-of-care in our setting. The positive role of routine use of artificial tears for effective reduction of postcataract surgery ocular discomfort was confirmed in other studies^36,37^.

Povidone iodine used before surgery for sterilization could possibly induce tear film instability and ocular surface damage^38^. However, both examined groups underwent the same perioperative procedures, with all patients receiving the same eyedrops and antiseptic immediately before the surgery. We must note that considering the availability of intracameral mydriatics/anaesthetics (e.g., Mydrane^®^), one could suspect less ocular surface distress at the time of surgery. Nevertheless, this would presumably affect both groups equally.

To the best of our knowledge, this is the first study to compare preservative-free and standard formulations of 0.1% dexamethasone in terms of dry eye signs and symptoms after standard cataract surgery. This study indicates and addresses possible postoperative complaints of the patients, such as foreign body sensation and treatment-related ocular surface impairment.

### Limitations to the study

The main limitation of the present study is the low number of eyes included in the analysis. Further studies with a larger sample size should follow. The second limitation is the lack of objective measures for ocular inflammation, such as laser flare photometry^39^. However, all subjective assessments were performed by one experienced clinician (MMW); therefore, grading bias is very limited^40^.

## Conclusions

In summary, using preservative-free dexamethasone caused milder discomfort and ocular surface staining at two weeks after cataract surgery compared to preserved dexamethasone. This calls for the routine use of nonpreserved eye drops after cataract surgery as a standard of care. The inflammation control comparison may require a larger study group.

### Supplementary Information

Supplementary Table 1.

Supplementary Table 2.

## Data Availability

Data are available upon request from Maria Muzyka-Woźniak at mmw@spektrum.wroc.pl.

## References

[1] Szakáts I, Sebestyén M, Tóth É, Purebl G (2017). Dry eye symptoms, patient-reported visual functioning, and health anxiety influencing patient satisfaction after cataract surgery. Curr. Eye Res..

[2] Garg P, Gupta A, Tandon N, Raj P (2020). Dry eye disease after cataract surgery: Study of its determinants and risk factors. Turk. J. Ophthalmol..

[3] Ishrat S, Nema N, Chandravanshi SCL (2019). Incidence and pattern of dry eye after cataract surgery. Saudi J. Ophthalmol..

[4] Miyake K, Yokoi N (2017). Influence on ocular surface after cataract surgery and effect of topical diquafosol on postoperative dry eye: A multicenter prospective randomized study. Clin. Ophthalmol..

[5] Choi YJ, Park SY, Jun I (2018). Perioperative ocular parameters associated with persistent dry eye symptoms after cataract surgery. Cornea.

[6] Naderi K, Gormley J, O'Brart D (2020). Cataract surgery and dry eye disease: A review. Eur. J. Ophthalmol..

[7] Kim JR, Oh TH, Kim HS (2011). Effects of benzalkonium chloride on the ocular surface of the rabbit. Jpn. J. Ophthalmol..

[8] Hedayatfar A, Hashemi H, Asgari S, Chee SP (2014). Comparison of efficacy and ocular surface toxicity of topical preservative-free methylprednisolone and preserved prednisolone in the treatment of acute anterior uveitis. Cornea.

[9] Pianini V, Passani A, Rossi GC, Passani F (2010). Efficacy and safety of netilmycin/dexamethasone preservative-free and tobramycin/dexamethasone-preserved fixed combination in patients after cataract surgery. J. Ocul. Pharmacol. Ther..

[10] Ylinen P, Holmström E, Laine I, Lindholm JM, Tuuminen R (2018). Anti-inflammatory medication following cataract surgery: A randomized trial between preservative-free dexamethasone, diclofenac and their combination. Acta Ophthalmol..

[11] Jee D, Park M, Lee HJ (2015). Comparison of treatment with preservative-free versus preserved sodium hyaluronate 0.1% and fluorometholone 0.1% eyedrops after cataract surgery in patients with preexisting dry-eye syndrome. J. Cataract Refract. Surg..

[12] Wolffsohn JS, Arita R, Chalmers R (2017). TFOS DEWS II Diagnostic Methodology report. Ocul. Surf..

[13] Schiffman RM, Christianson MD, Jacobsen G (2000). Reliability and validity of the Ocular Surface Disease Index. Arch. Ophthalmol..

[14] Whitcher JP, Shiboski CH, Shiboski SC (2010). A simplified quantitative method for assessing keratoconjunctivitis sicca from the Sjogren’s Syndrome International Registry. Am. J. Ophthalmol..

[15] Trusko B, Thorne J, Jabs D (2013). Standardization of Uveitis Nomenclature (SUN) Project. The Standardization of Uveitis Nomenclature (SUN) Project. Development of a clinical evidence base utilizing informatics tools and techniques. Methods Inf. Med..

[16] Cho Y, Kim MS (2009). Dry eye after cataract surgery and associated intraoperative risk factors. Korean J. Ophthalmol..

[17] Miura M, Inomata T, Nakamura M, Sung J (2022). Prevalence and characteristics of dry eye disease after cataract surgery: A systematic review and meta-analysis. Ophthalmol. Ther..

[18] Masafumi U, Takeshi K, Kusano M (2007). Acute corneal epithelial change after instillation of BAC evaluated using a newly developed in vivo cornea. Ophthalmic Res..

[19] Baudouin C, Brignole F, Becquet F (1997). Flow cytometry in impression cytology specimens: A new method for evaluation of conjunctival inflammation. Investig. Ophthalmol. Vis. Sci..

[20] Baudouin C, Hamard P, Liang H (2004). Conjunctival epithelial cell expression of interleukins and inflammatory markers in glaucoma patients treated over the long term. Ophthalmology.

[21] Baudouin C, Liang H, Hamard P (2008). The ocular surface of glaucoma patients treated over the long term expresses inflammatory markers related to both T-helper 1 and T-helper 2 pathways. Ophthalmology.

[22] Pisella PJ, Debbasch C, Hamard P (2004). Conjunctival proinflammatory and proapoptotic effects of latanoprost and preserved and unpreserved timolol: An ex vivo and in vitro study. Investig. Ophthalmol. Vis. Sci..

[23] Saettone MF, Giannaccini B, Monti D (2001). Ophthalmic emulsions and suspensions. J. Toxicol. Cutan. Ocul. Toxicol..

[24] Schoenwald RD, Stewart P (1980). Effect of particle size on ophthalmic bioavailability of dexamethasone suspensions in rabbits. J. Pharm. Sci..

[25] Sieg JW, Triplett JW (1980). Precorneal retention of topically instilled micronized particles. J. Pharm. Sci..

[26] Weijtens O, Schoemaker RC, Romijn FP (2002). Intraocular penetration and systemic absorption after topical application of dexamethasone disodium phosphate. Ophthalmology.

[27] Silverstein SM, Jackson MA, Goldberg DF, Muñoz M (2014). The efficacy of bromfenac ophthalmic solution 0.07% dosed once daily in achieving zero-to-trace anterior chamber cell severity following cataract surgery. Clin. Ophthalmol..

[28] Cagini C, Cometa F, Torroni G (2016). Dexamethasone disodium phosphate penetration into the human aqueous humor after topical application. Curr. Eye Res..

[29] Cagini C, Pellegrino A, Iannone A (2020). Comparison of anti-inflammatory effects of eye drops formulations after uncomplicated cataract surgery. Ther. Adv. Ophthalmol..

[30] Mikalauskiene L, Grzybowski A, Zemaitiene R (2021). Ocular surface changes associated with ophthalmic surgery. J. Clin. Med..

[31] Oh T, Jung Y, Chang D (2012). Changes in the tear film and ocular surface after cataract surgery. Jpn. J. Ophthalmol..

[32] Kohli P, Arya SK, Raj A (2018). Changes in ocular surface status after phacoemulsification in patients with senile cataract. Int. Ophthalmol..

[33] Zamora MG, Caballero EF, Maldonado MJ (2020). Short-term changes in ocular surface signs and symptoms after phacoemulsification. Eur. J. Ophthalmol..

[34] Ju R-H, Chen Y, Chen H-S (2019). Changes in ocular surface status and dry eye symptoms following femtosecond laser-assisted cataract surgery. Int. J. Ophthalmol..

[35] Yu Y, Hua H, Wu M (2015). Evaluation of dry eye after femtosecond laser-assisted cataract surgery. J. Cataract. Refract. Surg..

[36] Favuzza E, Cennamo M, Vicchio L, Giansanti F, Mencucci R (2020). Protecting the ocular surface in cataract surgery: The efficacy of the perioperative use of a hydroxypropyl guar and hyaluronic acid ophthalmic solution. Clin. Ophthalmol..

[37] Mencucci R, Boccalini C, Caputo R, Favuzza E (2015). Effect of a hyaluronic acid and carboxymethylcellulose ophthalmic solution on ocular comfort and tear-film instability after cataract surgery. J. Cataract Refract. Surg..

[38] Pels E, Vrensen G (1999). Microbial decontamination of human donor eyes with povidone-iodine: Penetration, toxicity, and effectiveness. Br. J. Ophthalmol..

[39] Liu X, McNally TW, Beese S (2021). Non-invasive instrument-based tests for quantifying anterior chamber flare in uveitis: A systematic review. Ocul. Immunol. Inflamm..

[40] Agrawal R, Keane PA, Singh J (2016). Comparative analysis of anterior chamber flare grading between clinicians with different levels of experience and semi-automated laser flare photometry. Ocul. Immunol. Inflamm..

