# Supplementary material for: Assessment of the eye surface and subjective symptoms after using 0.1% dexamethasone drops with and without preservatives in patients after cataract surgery
Source: Sci Rep. 2023 Oct 30;13:18625. doi: 10.1038/s41598-023-44939-1 (PMC10616106; doi:10.1038/s41598-023-44939-1)
Supplement: Supplementary file 1 — Supplementary Table 1. [file 41598_2023_44939_MOESM1_ESM.docx]

Supplementary Table S1. The description of symptoms provided for the subjects

|  | **Grade 0** | **Grade 1** | **Grade 2** | **Grade 3** |
| --- | --- | --- | --- | --- |
| **Itching** | No need to rub the eye | Occasional need to scratch | Frequent need to scratch | Constant need to scratch |
| **Discomfort** | None | Mild | Moderate | Severe |
| **Foreign body sensation** | None | Mild | Moderate | Severe |
| **Tearing** | Normal amount of tears | Increased amount of tears without tearing | Periodic tearing | Constant tearing |
| **Photophobia** | None | Slight difficulties in light requiring squinting | No significant difficulties requiring the wearing of sunglasses | Inability to withstand natural light even with sunglasses |
