# Supplementary material for: Assessment of the eye surface and subjective symptoms after using 0.1% dexamethasone drops with and without preservatives in patients after cataract surgery
Source: Sci Rep. 2023 Oct 30;13:18625. doi: 10.1038/s41598-023-44939-1 (PMC10616106; doi:10.1038/s41598-023-44939-1)
Supplement: Supplementary file 2 — Supplementary Table 2. [file 41598_2023_44939_MOESM2_ESM.docx]

Supplementary Table 2. Grading of anterior chamber flare and AC cells (SUN Workshop).^15^

| Grade/description of AC Flare | Grade of AC cells | Cells in field |
| --- | --- | --- |
| 0 none | 0 | <1 |
|  | 0.5+ | 1-5 |
| 1+ faint | 1+ | 6-15 |
| 2+ moderate (iris and lens details clear) | 2+ | 16-25 |
| 3+ marked (iris and lens details hazy) | 3+ | 26-50 |
| 4+ intense (fixed and plastic aqueous) | 4+ | 50+ |
